# STEPS-T Program Improves Endovascular Treatment Outcomes of Acute Ischemic Stroke; A 6-Year Study

**DOI:** 10.3389/fneur.2019.01251

**Published:** 2020-02-12

**Authors:** Ameer E. Hassan, Rani R. Rabah, Laurie Preston, Wondwossen G. Tekle

**Affiliations:** ^1^Department of Neurology, University of Texas Rio Grande Valley, Harlingen, TX, United States; ^2^Clinical Research Department, Valley Baptist Medical Center, Harlingen, TX, United States

**Keywords:** endovascular, acute ischemic stroke, recanalization, mechanical thrombectomy, quality initiative, clinical outcome

## Abstract

**Background:** Early endovascular recanalization of occluded vessels in acute ischemic stroke (AIS) is a major contributor to good clinical outcome. We report the analysis of all AIS patients throughout a 6-year experience following the deployment of a quality initiative aiming at improving care, speed and maintaining quality for AIS treatment.

**Methods:** Using a prospectively collected endovascular database at a comprehensive stroke center between 2012 and 2017, workflow/outcomes were recorded. There were no exclusion criteria. During the first year, a quality program employing “digital-object” technology, staff education, and workflow improvement was implemented to reduce time-to-treatment. Using electronic recording, workflow times were collected for onset (T_O_), CT (T_CT_), door (T_D_), angiography-suite (T_A_), groin puncture (T_G_), DSA (T_DSA_), and recanalization (T_R_). Recanalization time (T_G_-T_R_) and workflow intervals were compared at Year 1 and 6.

**Results:** Analysis of 382 patients (aged 71.3 ± 12.9) undergoing mechanical thrombectomy for AIS (206 male and 176 female) was performed. Recanalization time was significantly reduced from 82 min in 2012 to 34 min by 2017 (IQR 52–117 min and 23–49 min), a 59% reduction (*P* < 0.001). Further, consistent year-over-year reductions in setup time (T_A_-T_G_) (44% improvement) and T_CT_ to T_A_ times were observed. During the same period, clinical outcome significantly improved year-over-year as measured with the modified Rankin Scale 0–2 (33, 37, 38, 41, 53, and 58%).

**Conclusions:** Significant improvements were observed following the deployment of a quality initiative enabling iterative evidence-based process improvements, thereby sustaining significant reductions in time-to-treat and improved clinical outcomes for AIS patients.

## Introduction

Throughout the United States there is an acute ischemic stroke (AIS) occurring every 40 s and a stroke-related death occurring every 4 min, thus establishing stroke as the leading contender of morbidity and disability (1). Clinical outcomes, demonstrated by studies, are heavily reliant on reperfusion times (2, 3). In managing AIS cases due to large vessel occlusion, the goal has been to achieve rapid recanalization and to reduce time from onset to mechanical thrombectomy (MT) (4, 5). Optimal practices to generate improvement of effective and efficient case management remains immature and lacks a complete consensus, despite technological advancements in managing workflow.

Studies have demonstrated that modifications and improvements are potentially attainable at every stage of the care workflow, where considerable emphasis has been placed to reduce transport times (4, 6–16). The quality of care in AIS has been shown to be influenced by operational modifications such as the implementation of evidence-based stroke protocols, improved staff education and training, and prioritization of hospital resources (17, 18). The accumulation of these findings is in agreement with the necessity to improve efficiencies at individual stages of the care workflow, which is achievable through a methodologically incorporated alteration of the whole care pathway.

The main goal of this study is to determine the 6-year impact of STEPS-T (Stroke Triage Education and Procedure Standardization and Technology) on improving the time to treat, and thus determine whether clinical outcomes, measured using modified Rankin Scale (mRS), will show improvement in the study period, with a real world AIS population.

## Methods

### Patient Data Collection and Imaging Procedures

A prospectively maintained endovascular registry of patients treated with MT for AIS from 2012 to 2017 was obtained after institutional review board approval at Valley Baptist Medical Center Harlingen, a comprehensive, high-volume, stroke treatment center in the United States. The population included patients who presented with intracranial large-vessel occlusion (LVO) or distal occlusion, posterior circulation occlusions, or carotid occlusions and were eligible for MT after undergoing a standard computed tomography (CT) protocol [Alberta Stroke Program Early CT Score (ASPECTS) ≥6], confirmation of LVO, posterior circulation, or carotid occlusions on CT angiography, and absence of intracranial hemorrhage (ICH). Patients received intravenous (IV) tissue plasminogen activator (t-PA) after CT scan, when indicated (36% of the AIS patient population was administered an intravenous thrombolytic agent). All patients were imaged with CT and computed tomography angiography (CTA). All stroke thrombectomy patients were included, except nine cases (2%) had to be excluded during the data analysis due to missing data.

All procedures were performed by two Accreditation Council for Graduate Medical Education (ACGME) fellowship-trained neurointerventionalists. The following data were collected for each patient: demographic information, clinical characteristics, National Institutes of Health Stroke Scale (NIHSS) score at admission and discharge, modified Rankin Scale (mRS) at 90 days, and Thrombolysis in Cerebral Infarction (TICI) scores.

All procedures were performed using biplane angiographic systems (Innova IGS 630, GE Healthcare, Chicago, IL) equipped with 30 cm flat panel detectors and advanced imaging capabilities such as Cone Beam CT.

### Steps-T

**Figure 1:**
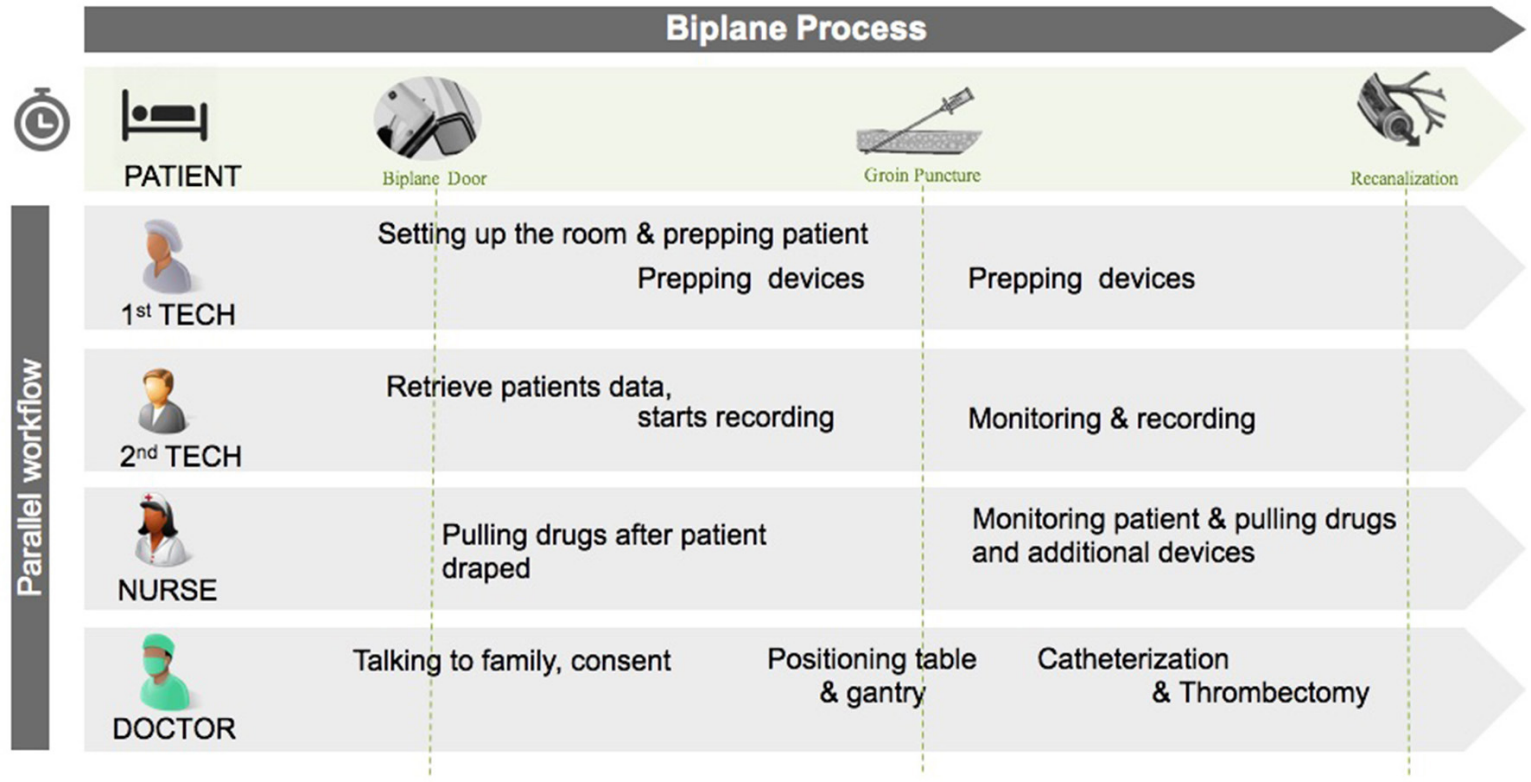
Workflow in the biplane suite illustrating the parallel nature of tasks by the different actors in the room. The “interventional time” starts when the patient arrives in the biplane suite. Tech, technician.

The STEPS-T program is an integrated approach designed to reduce door-to-recanalization time. The program fundamentally revolves around three interlaced principles affecting the speed and quality of care: (1) procedure workflow, (2) technologies, and (3) education. Additionally, each workflow step is recorded, using “digital objects,” or macro-enabled systems, which allow staff in the care pathway to remain connected, as stated by the American Heart Association Data Summit on the importance of integrating digital objects in modern stroke care (19, 20). Prior evidence indicates the importance of these three interrelated domains (15, 16, 18). These “digital objects,” custom built for our program by General electric (GE), were chosen to create time-stamps and to subsequently measure time metrics in the procedural workflow. The “digital objects” were captured manually by staff and housed in the Electronic Medical Records (EMR). By allocating workflow in the biplane angiography suite into steps, treatment time was reduced, and actions and actors of the intervention, i.e., nurses, technologists, and physicians, were able to operate in a timely and standardized fashion (Figure 1).

There were three stages involved in implementing STEPS-T at the comprehensive stroke center. Daily integrated and tailored formal training sessions were performed for the initial 30 days which included shadowing to customize imaging system settings and device training in order to improve their use. Thereafter advanced training was conducted monthly for 6 months to improve on weaknesses throughout the stroke treatment time periods and to optimize the work flow. Processes were later modified and amended, per clinical necessity, by the neurointerventionalist, nursing staff, technology provider and administration. Existing staff members were required to participate in the training, and to provide peer-to-peer training for ~3–6 months for all new staff who would shadow on-call procedures. Also, as new technology emerged staff was educated on its implementation as soon as possible.

Staff was briefed about the metrics monthly at Neuroscience Departmental meetings which were overseen by the two operating neurointerventionalists. Fine tuning and areas of improvement were discussed and identified through reports generated over time. Continuous modifications were made after identifying areas of potential improvement in treatment time and other areas, including changes in inclusion criteria after the DAWN study was initiated (21).

### Workflow Data

The use of digital objects (macros), appointed to each individual step in the care pathway, allowed for collection of the workflow data in a step-by-step manner. Using the Mac-Lab Hemodynamic Recording System (GE Healthcare, Chicago, IL) in the biplane suite, the times for each interventional/procedural step were recorded as digital objects. This permitted utilization of the customized step-by-step macro components for collection of time stamps, device(s) information, and medication administration(s). The overall times for each component in the care pathway were recorded and collected which include: patient onset time (T_O_), computed tomography time (T_CT_), arrival time at the center (door time, T_D_), arrival time at the angiography suite (T_A_), time of groin puncture (T_G_), acquisition of first digital subtraction angiography (DSA) (T_DSA_), and time of recanalization (T_R_).

### Statistical Analysis

Descriptive summaries were designated for each of the workflow intervals, including setup time (T_A_-T_G_), catheterization time (T_G_ until completion of T_DSA_ and T_M_), and clot retrieval time (T_M_-T_R_). The primary measures of total intervention (from T_A_ to T_R_) and recanalization time (from T_G_ to T_R_) were analyzed using a Wilcoxon rank-sum test. Workflow times and admission NIHSS scores were compared year to year from 2012 through the 6-year period using Kruskal-Wallis test. Results were classified as significant when *p* < 0.05.

## Results

We analyzed a total of 377 patients (mean age: 71.3 ± 12.9 years; 46% female), representing clinical characteristics common in an AIS population, who underwent MT. Of the 377 patients presenting with AIS throughout the 6-years, 240 patients (63.6%) presented with LVOs (anterior circulation), 41 patients (10.9%) presented with posterior circulation occlusions, and 96 patients (25.5%) presented with carotid occlusions necessitating angioplasty and or stenting in addition to MT (Table 1). The technique used in 95% of our cases involves deploying a stentriever, “kissing” the clot with a distal aspiration catheter, turning on suction, followed by pulling both the stentriever and distal aspiration catheter back together.

**Table 1:** Population characteristics.

**Data**	**Value**
*N*	377
Age in years, mean (std)	71.3 (12.9)
**Ethnicity** ***N*** (%)	
Hispanic	270 (71%)
White	104 (27%)
Asian	2 (1%)
African American	1 (1%)
Weight in kg, mean (std)	81.5 (20.4)
**Sex** ***N*** (%)	
Female	173 (46%)
Male	203 (54%)
**Comorbidities**	
Smoker	38 (10%)
Diabetes	176 (47%)
Hypertension	345 (92%)
Atrial fibrillation	119 (32%)
Coronary artery disease	117 (31%)
Congestive heart failure	40 (11%)
**Procedure by type:**	
Large vessel occlusion MT	240
Posterior circulation	41
Angioplasty (+MT)	96
**Site of occlusion:**	
Carotid terminus	111
MCA	207
ACA	17
BA/PCA	42
**Admission NIHSS, median (IQR)**	
2012	18 (13.5–23)
2013	18 (11–22)
2014	16 (11.5–21.5)
2015	16 (10–21)
2016	16 (10–21)
2017	18 (11.5–23)

*NIHSS, National Institute of Health Stroke Scale; MT, mechanical thrombectomy. MCA, Middle Cerebral Artery; ACA, Anterior Cerebral Artery; BA, Basilar Artery; PCA, Posterior Cerebral Artery*.

**Figure 2:**
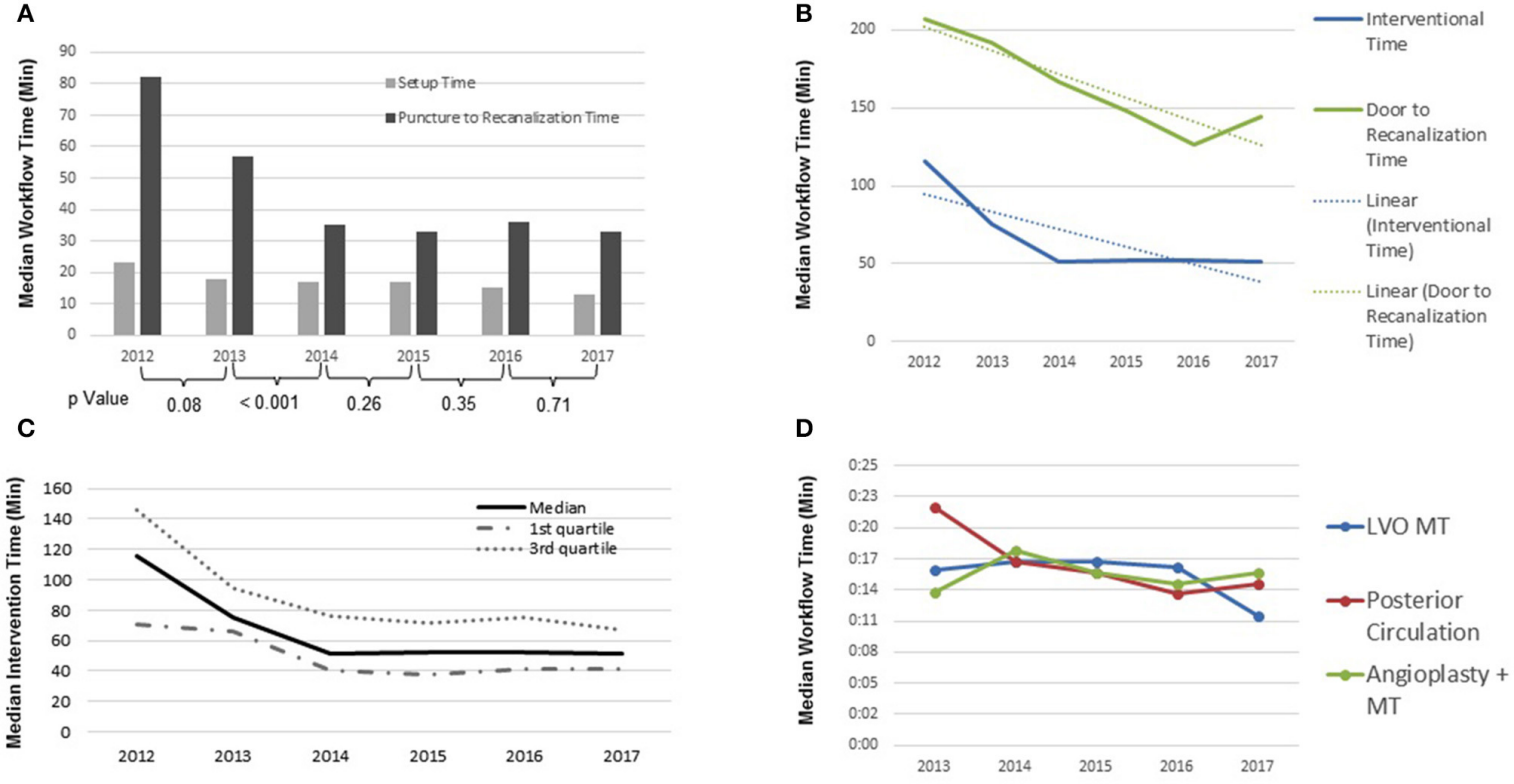
**(A)** Intraprocedural time: median setup time and puncture to recanalization time (*p*-values of individual years 2012–2017; 0.08, <0.001, 0.26, 0.35, 0.71); **(B)** periprocedural time: median interventional time and door to recanalization time 2012–2017 (Ratio of the linear regressions: *y* = −15.166x + 217.01, *R*^2^ = 0.8528/y = −8.8571x + 77, *R*^2^ = 0.6948); **(C)** intraprocedural time: median interventional time; **(D)** periprocedural time: recanalization time by type of procedure (LVO, large vessel occlusion; MT, mechanical thrombectomy).

Consistent year-over-year reductions in setup time (T_A_-T_G_) (23–13 min) were observed, representing a 44% improvement, accompanied by a reduction in T_CT_-T_A_ times (Figure 2). The median total intervention time (T_A_-T_R_) and door to recanalization time (T_D_-T_R_), were also significantly reduced from 116 min in 2012 to 51 min in 2017 and 207 min in 2012 to 144 min in 2017, respectively. Furthermore, intervention time remained constant throughout year 2014–year 2017 with a median of 51.5 ± 0.5 min, while door to recanalization time (T_D_-T_R_) experienced a constant decline throughout the 6-year period, with a slight increase in 2016 from 126 to 144 min in 2017. Approximately 75% (Ratio of the linear regressions: *y* = −15.166x + 217.01, *R*^2^ = 0.8528/y = −8.8571x + 77, *R*^2^ = 0.6948) of the total intra-hospital time reduction is attributed to the intra-biplane time (Figure 2).

Moreover, throughout the 6-year study period, median procedure time (TG–TR) for all AIS patients undergoing MT was significantly reduced from 82 min in 2012 to 34 min by 2017 (IQR 52–117 min and 23–49 min, respectively), representing a 53% reduction (*P* < 0.001) (Figure 2).

Since this study includes all AIS patients, recanalization time by procedure type (i.e., LVO MT, Posterior circulation, angioplasty + MT) was explored. The results displayed an overall reduction in both recanalization time for LVO and posterior circulation occlusion procedures from 2013 to 2017 (Figure 2).

Through 2013–2017, LVO MT patients remained somewhat constant with a median recanalization time of 16:41 ± 0.48 min, except between 2016 and 2017 where a significant reduction in recanalization time of 16.5–12 min can be appreciated (a reduction of 27.3%). Procedural recanalization time for posterior circulation occlusions display an overall reduction from 22 to 15 min (31.8% reduction) from 2013 to 2017. Recanalization times for patients requiring angioplasty (carotid occlusions) and MT exhibited an overall increase of 1.9 min (13.34%), with oscillations, from 2013 to 2017 (Figure 2).

**Figure 3:**
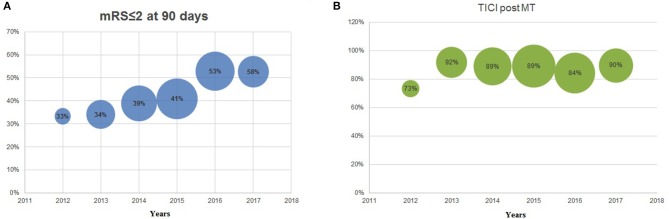
**(A)** modified Rankin Scale (mRS) ≤2 at 90 days 2012–2017; **(B)** thrombolysis in cerebral infarction (TICI) post-mechanical thrombectomy 2012–2017. The size of the bubble is proportionate to the sample size per year.

**Graph 1:**
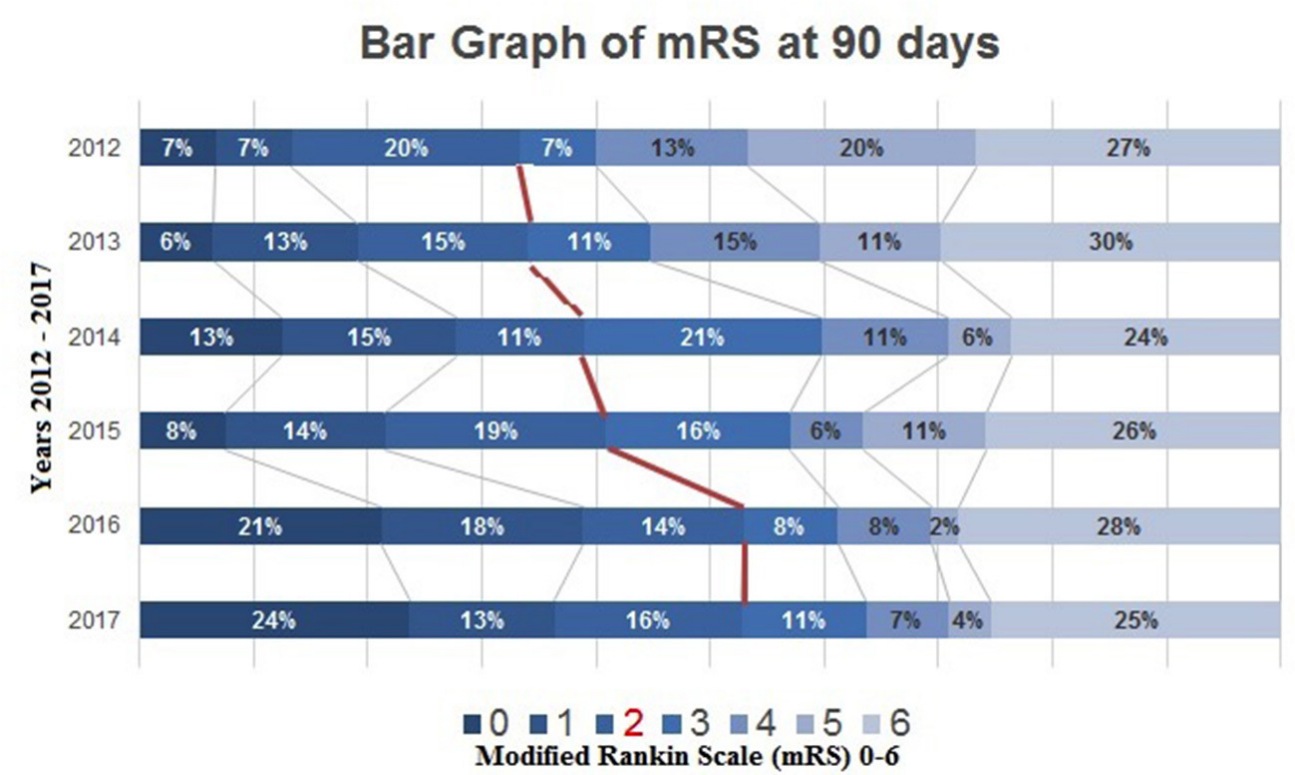
Bar graph of modified Rankin Scale (mRS 0–6) at 90 days post-mechanical thrombectomy.

During the same 6-year study period, clinical outcome also significantly improved year-over-year as measured with the modified Rankin Scale (mRS) 0–2 at 90 days, evolving from 33% during 2012, to 37, 38, 41, 53, and 58% during the following years. Analyzing the last 3-years together, 90-day mRS of 0–2 was equal to ~50% (Figure 3). In addition to the patients with an mRS of 0–2 at 90 days, patients with an mRS 3–6 were also analyzed throughout years 2012–2017, giving a more holistic representation of the AIS population (see Graph 1).

There was no significant difference in recanalization rates (TICI 2b-3) throughout the 6-years, but an increase can be appreciated between 2012 and 2013 (73–92%, NS) (Figure 3). In the same 6-year period, the median admission NIHSS remained stable (18, 18, 16, 16, 16, 18) along the successive years for all AIS patients (Table 1).

## Discussion

Our analysis of AIS patients during the 6-year period included patients that presented with anterior LVOs, posterior LVOs, and tandem lesions (requiring initial angioplasty followed by MT). There were no exclusions, thus depicting a tangible representation of the “real world” AIS population. The accumulative analysis of these patients, generated the following summarized results: a decrease in setup (T_A_-T_G_), median total intervention (T_A_-T_R_), and door to recanalization time (T_D_-T_R_) of 44, 56, and 30.4% respectively through 2012–2017, a total median procedure time (T_G_-T_R_) reduction of 53%, sub-classified by procedure type [LVO (T_G_-T_R_) reduced by 27.3%, posterior circulation occlusion (T_G_-T_R_) decreased by 31.8%, while angioplasty and MT (T_G_-T_R_) were increased by 13.34%], mRS improvement from 33% in 2012 to 58% in 2017, recanalization rates (TICI 2b-3) showed an overall increase from 73% in 2012 to 90% in 2017, and no significant difference in demographics and admission NIHSS scores throughout the 6-years.

Every 30-min delay in angiographic reperfusion reduced the relative likelihood of a good clinical outcome (mRS 0–2 at 90 days) by 15% (22). Prior studies have investigated the predictors of good clinical outcome after endovascular treatment for AIS and the need to reduce time-to-treatment. The need for implementation of workflow techniques to reduce procedural times are highlighted in a study by Settecase et al. (23), where the integration of a stroke cart (containing all procedural MT equipment), parallel staff workflows, and use of conscious sedation reduced in-room time to groin puncture (−21.3 min, *p* < 0.0001), in-room to on-clot time (−24.1 min, *p* = 0.001), and in-room to reperfusion time (−29.5 min, *p* = 0.01) for 47 patients (19 pre- and 28 post-implementation) with LVO who underwent MT (23). In our analysis, we included additional procedural types (posterior circulation occlusions and tandem lesions) and reached a similar consensus with a reduction in setup (T_A_-T_G_) and procedure (T_G_-T_R_) time (−10 and −48 min, respectively) throughout the 6-years.

In an additional study, reductions were centralized around door to groin-puncture time (DGPT) for 16 LVO patients (throughout 6 months) who underwent MT after implementation of time reduction techniques. The conclusion was that “An iterative quality improvement process can significantly improve DGPT” (24). Thus, reductions at different stages of the care pathway are possible, and are in concurrence with generating a more favorable outcome. Also, the HERMES analysis (Highly Effective Reperfusion Evaluated in Multiple Endovascular Stroke Trials) reported a linear reduction in chances of good outcome with increasing time between onset and MT (17).

Overall, total intervention, recanalization, and setup time were reduced in this 6-year period. This study highlights the importance of time-to-treatment by endovascular therapy and the role an integrated program (STEPS-T), implemented to reduce time-to-treatment, plays in the AIS picture. Time-to-treatment is an important metric to assess quality improvements to workflow and has proven to directly affect stroke outcomes (12).

This study demonstrated that total intervention and recanalization times in all stroke patients was reduced over the 6-year period by implementation of the STEPS-T program and through the experience of the operators and staff. Total intervention time was reduced by 56%, recanalization time by 30.4%, and setup time by 44%. mRS, as an outcome, was also improved from 33 to 58%. Moreover, STEPS-T sustainably reduced times along every stage of the care pathway.

Even though much attention has been placed on reducing patient transfer time to shorten the overall care pathway, manipulation of treatment duration, not at the expense of quality care, has also been demonstrated. A harmonized and consistent approach has been reported, by McTaggart et al., to reduce time from groin puncture to recanalization (4). The McTaggart et al. study was conducted over a 1-year observational period of a smaller cohort (*N* = 22, mean 37 min, CI: 32.1–42.5), excluding posterior circulation and tandem lesion cases, and clinical outcomes were not reported at 90 days.

Limitations to this study included the retrospective nature of data collection and analysis that prevented randomization of the approach. Also, detailed analysis was not performed to assess the evolution from the time of onset to treatment over the 6-year period. Due to the DAWN trial (clinicaltrials.gov number, NCT02142283) criteria there was inclusion of wake-up strokes exceeding the 6-h window, which slightly affected the 2016–2017 data, however patients who underwent MT throughout 2012–2016 were free from any inclusion or exclusion criteria. The 2016 data witnessed increased AIS patient recruitment, following publications of studies that proved superiority of MT to previously established standard of care (5, 7, 25–27). Ultimately leading to an abnormally increased staff turnover in 2016, affecting our demanding training approach. Despite the lack of development to our practice, we appreciated a reduction in time and an improvement in operator skills performing MT, which is likely due to the increase in AIS patients throughout the study independent of the STEPS-T application. Also, it is worth noting is that 95% of our cases were performed using stent-retriever with aspiration. STEPS-T implemented in a lower-volume center may prove more challenging and time consuming, necessitating multi-center sharing and standardization (28). Some barriers to the implementation to STEPS-T included initial staff biases, anxiety, and the concern over increased workload. Continuous staff training, shadowing, and education helped to improve these issues over the course of the first couple of months. Increased MT volume facilitated implementation of the program. Support from hospital education and industry further refined their skills. For new hires a minimum of 3 months shadowing on call helped transition them to reach the program's goals. The future of stroke treatment will require new education initiatives to improve the onset to door and door to catheterization lab times to continue improvements in stroke outcomes.

## Conclusion

STEPS-T continues to prove beneficial in reducing total intervention and recanalization time, consequently generating an improvement in mRS, even when including “real world” patients presenting with posterior circulation and tandem lesions (angioplasty followed by MT). Further standardization of digital objects and staff training will be required to continually sustain the evidence-based improvements at all steps of the acute stroke care pathway.

## Data Availability Statement

The datasets generated for this study are available on request to the corresponding author.

## Ethics Statement

Following protocol approval by Valley Baptist Medical Center-Harlingen and MetroWest Medical Center-Framingham institutional review boards, written and informed consent was obtained from patients enrolled in the DAWN trial (clinicaltrials.gov number, NCT02142283). This patient population was included in the endovascular registry of patients treated with MT for AIS throughout 2016-2017.

## Author Contributions

AH implemented the quality initiative and, along with WT, performed the neurointerventional procedures. AH, RR, LP, and WT were involved in data abstraction, analysis, and interpretation. AH and RR took the lead in writing the manuscript, while AH, RR, LP, and WT contributed in drafting and revising the manuscript. All authors approved the final draft for submission and agreed to be accountable for all aspects of the work involved to produce this manuscript.

### Conflict of Interest

The authors declare that the research was conducted in the absence of any commercial or financial relationships that could be construed as a potential conflict of interest.
